# Parapharyngeal Abscesses Caused by Group G Streptococcus

**DOI:** 10.1155/2018/7307290

**Published:** 2018-09-27

**Authors:** Shori Tajima, Takashi Anzai, Rina Matsuoka, Hiroko Okada, Takuma Ide, Mitsuhisa Fujimaki, Shota Kaya, Shin Ito, Katsuhisa Ikeda

**Affiliations:** Department of Otorhinolaryngology, Juntendo University Faculty of Medicine, Tokyo, Japan

## Abstract

Deep neck abscess is a life-threatening infection that causes laryngeal edema and upper airway occlusion. The predominant bacterial species involved in this disorder is group A streptococcus. Group G streptococcus (GGS) constitutes the normal commensal flora of the human upper airway. Although rarely, it can cause pharyngitis, tonsillitis, and peritonsillar abscess. Here, we report a case of a woman with parapharyngeal abscess caused by GGS. A 56-year-old woman presented to the emergency department with complaints of sore throat and cervical swelling, and a diagnosis of parapharyngeal abscess was established. She had upper airway occlusion, requiring urgent tracheostomy. Endoscopic incision and drainage of the abscess using a specially designed, rigid curved laryngoscope was successfully performed. Since a rigid curved laryngoscope creates a wide viewing field and working space, it was useful for incision and drainage of the parapharyngeal abscess.

## 1. Introduction

Deep neck abscess, including parapharyngeal and retropharyngeal abscesses, is a life-threatening infection that can cause laryngeal edema and upper airway occlusion. Several case reports showed that group G streptococcus (GGS) bacteria are normal commensal flora of the human upper airway that can cause severe pharyngitis, tonsillitis, and, rarely, peritonsillar abscess [1–3]. Here, we present a case of parapharyngeal abscess with upper airway occlusion caused by GGS. We successfully performed endoscopic surgery for incision and drainage of the abscess using a rigid curved laryngoscope that is a specially designed laryngoscope developed by Satou (Satou's Curved Laryngo-Pharyngo Scope®; Nagashima Medical Instruments Company, Tokyo, Japan).

### 1.1. Case Presentation

A 56-year-old woman presented to our emergency department with complaints of sore throat and cervical swelling. Her medical history included hypertension, habitual smoking, and occasional alcohol consumption. She developed a sore throat and visited a local clinic 3 days before presenting to our emergency department.

She was diagnosed with tonsillitis. Group A antigen test was negative, and she was administered amoxicillin 750 mg/day. However, 2 days later, she developed dyspnea, dysphagia, and neck stiffness. On arrival at our hospital, she had a severe sore throat and muffled voice and was drooling. Laryngeal fiberscopy revealed swelling of the caudal oropharyngeal mucosa on the right side and a severely swollen epiglottis and arytenoid region that caused upper airway occlusion. Her SpO_2_ was 97% on 2 L oxygen, and her body temperature was 37.4°C. Blood test results suggested strong inflammation (white blood cell count, 15.3 × 109/L; C-reactive protein, 27.6 mg/L).

**Figure 1:**
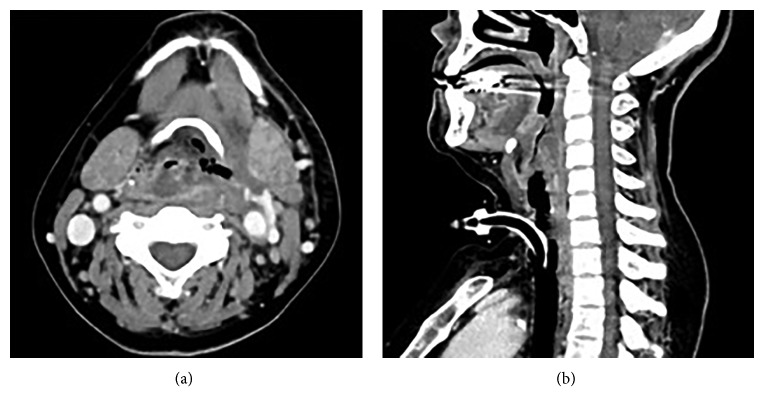
Enhanced CT findings (after tracheostomy). Enhanced CT scan revealed a large mass involving the left parapharyngeal and retropharyngeal space, causing significant airway narrowing.

We established a diagnosis of parapharyngeal abscess. Because of a high risk of suffocation, we first performed tracheostomy with the patient under local anesthesia. Enhanced computed tomography after tracheostomy revealed hypodense lesions at the left lateral and posterior pharyngeal walls (Figure 1). Incision and drainage of the abscess was performed with the patient under general anesthesia using a rigid curved laryngoscope.

**Figure 2:**
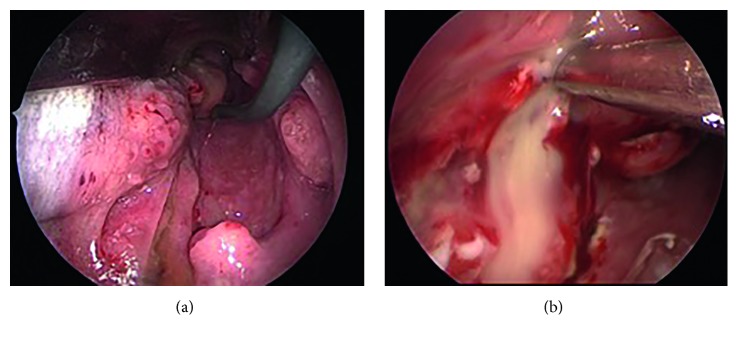
Intraoperative findings. (a) There was peritonsillitis containing mucus and pus from the posterior pillar on the right side. (b) Incision at the left lateral and posterior pharyngeal walls. A large amount of pus flowed out from the peritonsillar and lateral pharyngeal wall.

Peritonsillitis containing mucus and pus from the posterior pillar was observed (Figure 2(a)). We incised and opened a part of the swollen posterior pillar and lateral and posterior pharyngeal walls, draining pus from these regions (Figure 2(b)). The operation was completed without any adverse events.

The patient was administered 3 g/d meropenem as empiric therapy. On postoperative day 4, culture for aerobes and anaerobes revealed GGS and *Parvimonas micra*, respectively. Therefore, the antibiotics were changed to 4 g/d piperacillin and 1.2 g/d clindamycin. The recovery course was uneventful.

## 2. Discussion

GAS is a major microbial pathogen causing pharyngitis, peritonsillar abscess, and deep neck infection. In our case, GGS and *Parvimonas micra* were isolated from the pus. It is often the case that mixed aerobic and anaerobic bacteria can be identified through pus culturing. Tsai et al. reported that polymicrobial growth was observed in 57.39% of pus cultures [4]. Empirical antibiotics targeting both aerobes and anaerobes would be appropriate. However, considering GGS's aggressive characteristic like GAS, GGS appeared to be an important pathogen of the parapharyngeal and concomitant peritonsillar abscesses that could cause potentially fatal upper airway occlusion. GGS is frequently present in the human pharynx and tonsils. Group C and G streptococci are two antigenic variants of the same organism, *Streptococcus dysgalactiae* subspecies *equisimilis* (SDSE). Genomic sequence homology analysis of GGS revealed that GGS was closest in sequence to GAS, with 72% similarity [5]. Virulence profile analysis of SDSE revealed that its genetic basis of disease propensity is shared with GAS, including the antiphagocytic M protein, streptolysin O, streptolysin S, streptokinase, and one or more pyrogenic exotoxins [6]. The burden of SDSE infection is comparable to that caused by invasive GAS infection [7]. We reviewed the bacteriology of the peritonsillar abscess from previous studies searching the PubMed database. There were 9 studies from 2014 to 2018. Two studies were excluded as the detailed *Streptococcus* sp. was not mentioned. As shown in Table 1, GGS or GCS has a prevalence of less than 5% [8–14]. Notably, GGS was not detected using the rapid antigen test because of the lack of group A antigen, which is the target of these tests. Current pharyngitis guidelines focus only on group A streptococci and only recommend antibiotics. However, as we demonstrated, GGS also causes life-threatening diseases, such as a deep neck abscess. It is important for the primary care physician to consider carefully negative results.

**Figure 3:**
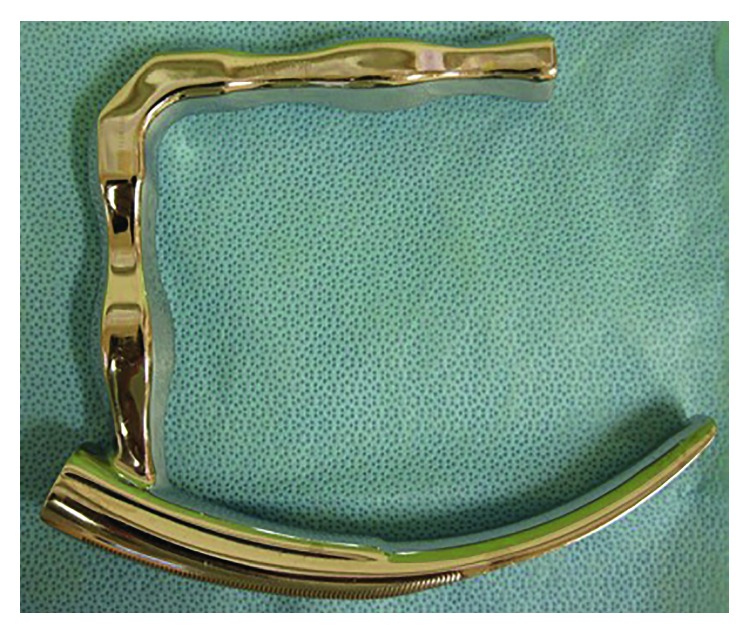
A rigid curved laryngosocpe. This curved rigid laryngoscope can enable visualization of the whole oropharynx.

During surgery, it is important to visualize the abscess lesion and create a sufficient working space. The intraoral approach using a self-retaining mouth gag with ipsilateral tonsillectomy can be used to access diseases in the parapharyngeal space [15]. However, a Davis gag can be used to visualize oropharyngeal structures around the oral cavity and tonsils but cannot be used to visualize the caudal oropharynx. Therefore, physicians should perform invasive surgery to remove the ipsilateral tonsil and approach the parapharyngeal space. We describe a novel method using a rigid curved laryngoscope for incision and drainage of a parapharyngeal abscess. This instrument was designed for laryngopharyngeal surgery under endoscopic vision [16, 17] (Figure 3). Recent case reports have described successful removal of a fish bone in the hypopharynx and drainage of retropharyngeal abscesses using a rigid curved laryngoscope [18, 19], which is useful for hypopharyngeal and oropharyngeal surgery. The blade is inserted into the pharynx and lifted forward. The oropharynx is well visualized. After exposing the whole oropharynx, the handle is attached to a holder fixed to the operating table. Because of a crooked line path, we used devices such as malleable high-frequency knives (KD-600®; Olympus, Tokyo, Japan) and malleable forceps (Laryngo FIT®; Karl Storz, Tuttlingen, Germany). In our procedure, the rigid curved laryngoscope exposed the whole oropharynx including the caudal oropharynx. A conventional straight laryngoscope can be used to visualize the caudal oropharynx; however, it provides only a small visual and working space. This curved instrument can provide a wide working space that permits easy and complete incision and drainage while avoiding neck incision or tonsillectomy; therefore, it is useful for incision and drainage of a parapharyngeal abscess.

## 3. Conclusion

GGS is an important pathogen of deep neck abscesses. A rigid curved laryngoscope enables a wide viewing field and working space; therefore, it is useful for incision and drainage of a parapharyngeal abscess.

## Figures and Tables

**Table 1:** Previous studies involved in bacteriology of peritonsillar abscess.

Investigator	Country	Year	Number of samples, *n*	GGS or GCS, *n* (%)
J. Wiksten et al. [8]	Finland	2010-2011	149	2 (1.3)
Vaikjarv et al. [9]	Estonia	2011-2012	22	1 (4.5)
Tachibana et al. [10]	Japan	2008–2013	100	1 (1.0)
Lepelletier et al. [11]	French	2009–2012	412	11 (2.6)
Plum et al. [12]	USA	2002–2012	69	1 (1.4)
Mazur et al. [13]	Poland	2003–2013	45	1 (2.2)
Gavriel et al. [14]	Israel	1996–2003	132	1 (0.76)

GGS, group G streptococcus; GCS, group C streptococcus.
